# P190: Surgical site infection surveillance system in São Paulo state, Brazil

**DOI:** 10.1186/2047-2994-2-S1-P190

**Published:** 2013-06-20

**Authors:** DS Mello, MC Padoveze, G Madalosso, SA Ferreira, DBD Assis

**Affiliations:** 1 Collective Health Nursing Department, School of Nursing, University of Sao Paulo, São Paulo, Brazil; 2 Division of Infection Control, Health State Department, São Paulo, Brazil

## Introduction

Governmental authorities should establish priorities for surveillance and define what kind of information to be gathered from healthcare facilities. Many developing countries cannot afford to manage Surgical Site Infection Surveillance System (SSISS) that includes all types of surgeries; therefore a criterion should be used to select the best indicator to be monitored. Since 2004, the SSISS in São Paulo have been focused only crude rates of Surgical Site Infections (SSI) in clean surgeries.

## Objectives

The present study aimed to select and to implement indicators for a new SSISS in the State.

## Methods

Mixed method design including a methodological study and a prospective descriptive study, carried out from July 2011 to September 2012, in three phases: 1) Methodological study carried out by means of literature review and expert validation aiming to identify the best criteria for selection of SSI indicators to be monitored; 2) Implementation of a new SSISS; 3) Follow-up of 6 months after implementation. The participating hospitals (n=306) represented 38.3% of total acute care hospitals in the State.

## Results

The main criteria identified to select the SSI indicators were: a) magnitude of the surgery in the Brazilian Universal Health System; b) severity of harm in case of SSI; c) potential impact of prevention strategies; d) recommendation by federal normative; d) potential for benchmarking against at least other three SSISS worldwide. Outcome indicators of SSI for the following surgeries were selected: cesarean section, hip and knee arthroplasty, CABG, craniotomy, mastectomy, and laparoscopic procedures: cholecystectomy, herniorrhaphy, hysterectomy, appendectomy, and colectomy. The SSI rates identified (3^rd^ quartiles) and the number of surgeries monitored in the period were respectively: 0.9 (n=75816), 2.7 (n=2305), 0.0 (n= 2477), 8.0 (n=1949), 1.2 (n=1789), 0.0 (n=1702), 0.0 (n=13332), 0.0 (n=4904), 0.0 (n=1549), 0.0 (n= 1287), 0.0 (n=364).

## Conclusion

The development of criteria supported the rational selection of indicators for governmental monitoring of SSI. Despite good adherence to the project, data suggest that SSI may be underestimated. A longer period of evaluation will be performed next. Efforts should be focused on the improvement of data quality for SSISS.

## Disclosure of interest

None declared

